# Improvement in functional gait parameters following corrective thoracolumbar surgery in children affected by Mucopolysaccharidosis 1 (Hurler syndrome)

**DOI:** 10.1186/s13023-020-01427-3

**Published:** 2020-06-05

**Authors:** Rajkumar Sundarapandian, Simon Jones, Alexander Broomfield, Pauline Hensman, Neil Oxborrow

**Affiliations:** 1 Department of Spinal Surgery, Salford Royal Hospital, Stott lane, Salford, United Kingdom; 2 Department of Spinal Surgery, Royal Manchester Children’s Hospital, Oxford Road, Manchester, United Kingdom; 3 Manchester Centre for Genomic Medicine, Royal Manchester Children’s Hospital, Oxford Road, Manchester, United Kingdom

**Keywords:** Mucopolysaccharidosis 1, Gait, Kyphosis, Spinal surgery, Functional outcome

## Abstract

**Objective:**

Thoracolumbar kyphosis is a common indication for spinal surgery in children with Mucopolysaccharidosis. Functional outcome of spinal surgical intervention has never been published in patients with this rare disease. We present a cohort of patients with Mucopolysaccharidosis 1(Hurler syndrome) who underwent thoraco-lumbar spinal deformity correction and functional outcome assessed by pre-operative and post-operative gait analysis. This study represents the first attempt at presenting a functional assessment of surgical outcome in any Mucopolysaccharidosis subtype.

**Methods:**

A retrospective analysis of prospectively collected data was carried out from 11 children diagnosed with this subtype of Mucopolysaccharidosis. All patients underwent thoracolumbar kyphosis correction between the years 2013 to 2016. Gait assessment was performed using GAITRite™ electronic walkway pre-operatively and post-operatively within 9 to 24 months from the index surgery. Walking distance, cadence and gait velocity were the three spatio-temporal parameters analysed. Wilcoxon signed rank test was used to analyse the data and *P*-Value ≤0.05 was deemed significant.

**Results:**

There was a statistically significant improvement in walking distance in 9 out of 11 patient post-operatively with a mean increase of 232.06 cms (*P* = 0.05). There was marginal improvement in cadence by 6.33 steps/min post-operatively (*P*-value 0.79). Gait velocity also showed a marginal increase by 8.73 cms/sec post-operatively (*P*-value 0.32).

**Conclusion:**

The results of our study suggest that correction of thoracolumbar kyphosis in children with Mucopolysaccharidosis 1 resulted in a significant improvement of walking distance with a trend towards improved gait in the other parameters. Post-operative change in cadence was not statistically significant suggesting that physiological maturation of gait had minimal effect in the specified post-operative assessment timeframe. This study emphasizes that outcomes of spinal surgery in children with Mucopolysaccharidosis 1 should be determined by functional measures aiming to maintain or improve quality of life.

## Introduction

Mucopolysaccharidosis 1(MPS1) or Hurler syndrome is a rare autosomal recessive disorder which results in a deficiency of lysosomal enzyme α-L-iduronidase [1]. This leads to failure in the degradation of glycosaminoglycans and their accumulation in various systems of the body. The global incidence of MPS 1 was reported as 1 in 100,000 live births [2]. The incidence of MPS 1 in England and Wales was 1.07 per 100,000 live births [3].

The commonest skeletal manifestations are thoracolumbar kyphosis, hip dysplasia, genu valgum [4] and these persist despite disease modifying therapies [5]. MPS 1 exists on a spectrum with severe mutations in IDUA leading to complete enzyme deficiency and more rapidly progressive disease. Standard of care for this phenotype of MPS 1 is Haematopoietic stem cell transplantation (HSCT).

**Fig. 1:**
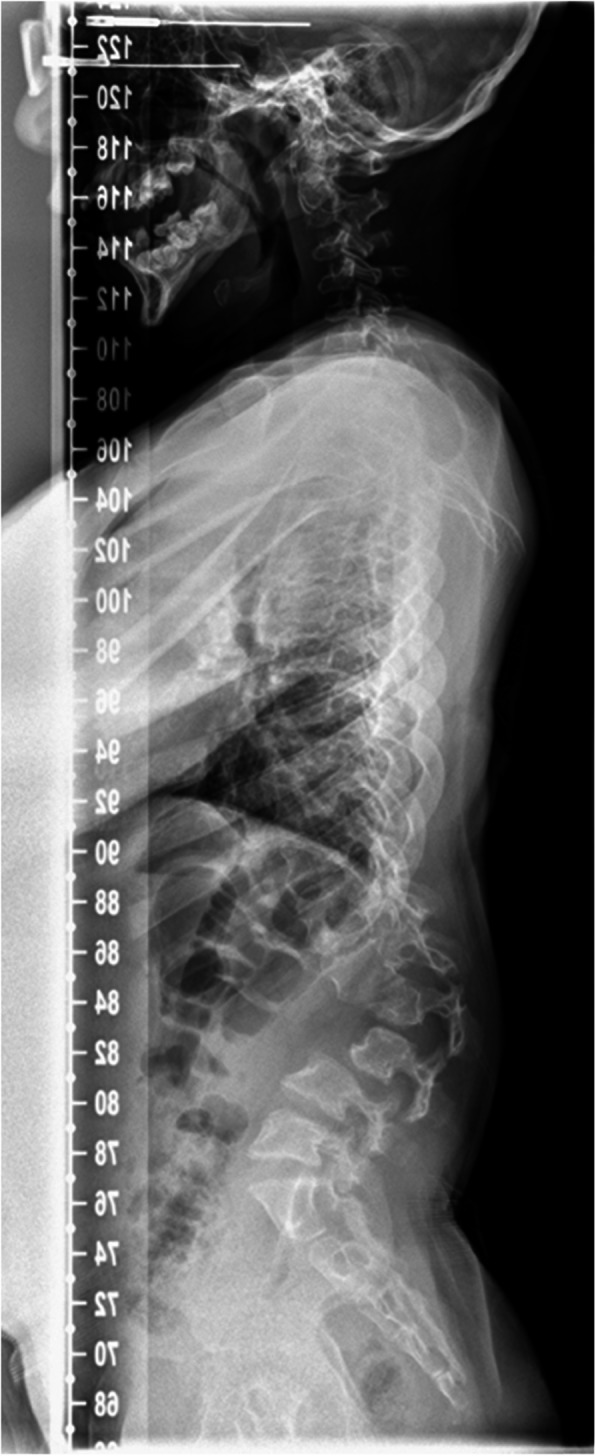
Pre-operative radiograph of thoracolumbar spine showing high lumbar kyphosis- Lateral view

**Fig. 2:**
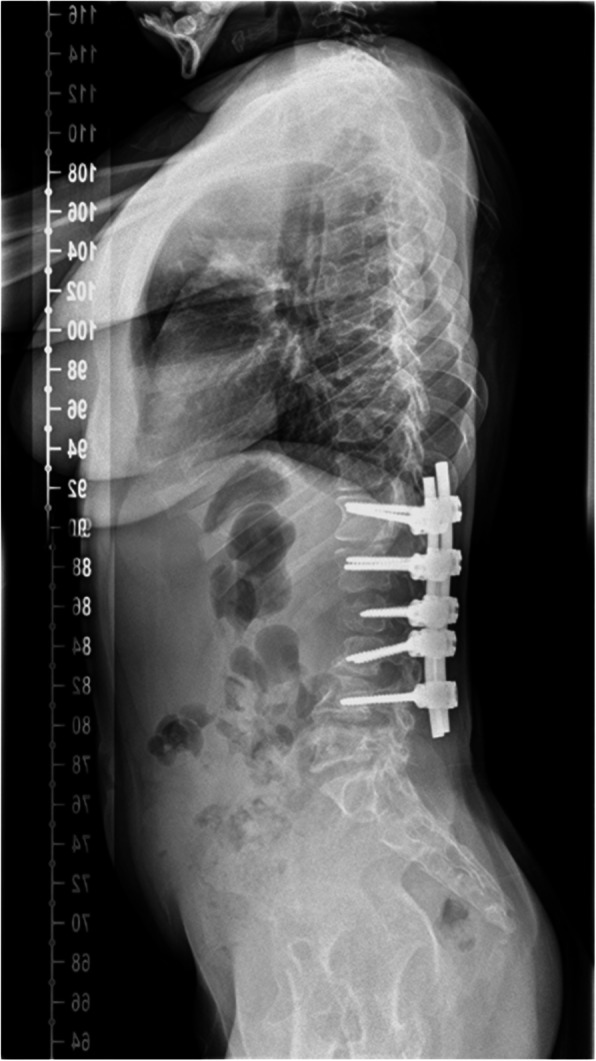
Post-operative radiograph of the same patient after correction of thoracolumbar kyphosis- Lateral view

Surgical interventions to address dysostosis multiplex in this cohort of patients are associated with high risk and must be justified on clinical grounds and clear indication. Functional outcome measures should determine the overall response to surgical intervention, yet reported outcomes in literature commonly focus on radiological measures. There are no studies which report functional outcome before and after spinal surgery for thoracolumbar kyphosis [6]. Radiological outcome alone in insufficient to assess functional improvement after corrective spinal surgery in this cohort of patients. Surgery to correct thoracolumbar kyphosis is indicated considering the progression of deformity and its flexibility, presence of symptoms, growth potential and co-morbidities [7] in this group of patients. We present a retrospective cohort study of homogenous patients with severe MPS 1 post HSCT who underwent correction of thoracolumbar kyphosis (Fig. 1 and Fig. 2) and functional outcome assessed by pre-operative and post-operative gait analysis. This study represents the first attempt at presenting a functional assessment of surgical outcome in any MPS subtype.

### Methodology and research design-

The objective of our study was to assess gait using validated measures in children with MPS1 before and after correction of thoracolumbar kyphosis. There are over 70 patients with MPS 1 being followed up in the MPS clinics and radiographs to assess thoracolumbar kyphosis are performed during each visit. Surgical intervention is performed on those patients with progressive thoracolumbar kyphosis or with loss of flexibility of the deformity. Patients with progressive deformity also undergo serial MR scans (Magnetic resonance imaging) in supine position and persistent or irreducible kyphosis in supine imaging was deemed as stiff deformity which was surgically intervened. There was bladder dysfunction in one patient with significant thoracolumbar kyphosis who was taken up for urgent decompression with correction of the deformity. Bladder function returned to normal following the surgery.

The selection criteria for surgery were patients with MPS1 and thoracolumbar kyphosis with progressively worsening deformity or with stiffening deformity or with impending neurological deficit. The gait data of these patients who underwent surgery between the time period of 2013 to 2016 were retrospectively analysed. All the other patients with MPS1 being followed up were not taken up for surgery as the deformity was not progressive. Patients of other subtypes of MPS were also excluded. A thorough pre-operative assessment and optimisation was done by a highly skilled multi-disciplinary team of clinicians including a specialized Paediatric anaesthetist when surgery was indicated. All the children who underwent correction of thoracolumbar kyphosis were operated by a single senior Paediatric Spinal Surgical Consultant.

A retrospective analysis of prospectively collected gait data was undertaken of MPS1 patients who underwent thoracolumbar kyphosis correction between the years 2013 to 2016 in our centre. A total of 11 patients were identified and included in the study, out of which 9 patients had single stage Posterior spinal deformity correction and 2 patients had undergone Anterior release followed by Posterior spinal deformity correction. All patients had completed atleast 9 months following surgery and were capable of walking independently.

There were 5 male and 6 female patients, aged 5–13 years (mean 9.27 years) at the time of surgery in the study group (Table 1). The post-operative gait assessment in months from the index surgery ranged from 11 to 35 months (mean 18.45 months).

**Table 1:** Patient demographics with age at time of stem cell transplant, age at time of surgery and month at which post-operative gait assessment was done

Patient Identifier	Gender	Age at the time of transplant in months	Age at the time of surgery in years	Post-operative gait assessment in months after surgery
1.	Male	15 months	10 years	28 months
2.	Female	10 months	9 years	14 months
3.	Female	1st- 11 months 2nd- 16 months	11 years	13 months
4.	Female	1st- 11 months 2nd- 19 months	13 years	16 months
5.	Female	12 months	10 years	18 months
6.	Female	9 months	11 years	16 months
7.	Female	14 months	9 years	11 months
8.	Male	8 months	5 years	24 months
9.	Male	12 months	7 years	17 months
10.	Male	8 months	7 years	35 months
11.	Male	20 months	10 years	11 months

### Gait assessment

Gait analysis was performed using the GAITRite™ system during each clinic visit.

**Fig. 3:**
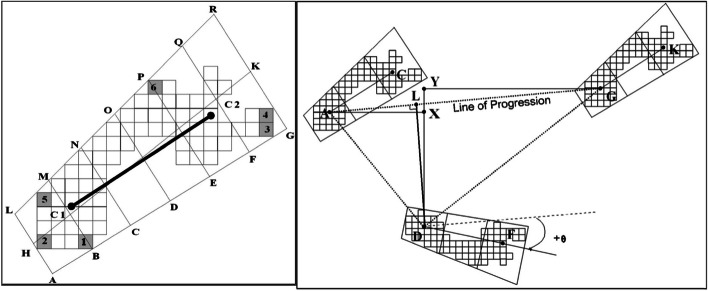
Identification of quadrilaterals, midline of foot and the line of progression using the walkway- Image reproduced with permission from CIR Systems, Inc. GAITRite Electronic walkway technical reference (WI-02-15. Rev. L 5/6/2013)

Each child was made to walk the length of the walkway back and forth, without footwear or orthosis at a comfortable pace for one minute. GAITRite™ electronic walkway is a mat embedded with sensor pads to measure spatial and temporal parameters of gait. Multiple interconnected sensor pads form the length of the walkway on which subjects walk. The pressure sensitive sensors activate to sense geometry of the object, relative arrangement between them in a two-dimensional space and vertical component of the pressure exerted by the object. When the subject walks across the walkway (Fig. 3), footprints are obtained as quadrilaterals in real time with information on relative arrangement with the opposite feet [8]. Each walk of the subject is recorded in a laptop which is connected to the walkway. We analysed cadence, gait velocity and walking distance of all the children included in the study group.

The closest pre-operative assessment was selected along with the post-operative assessment recorded between 9 to 24 months. The latter time frame was chosen to allow recovery from surgery and also minimise any potential confounding progression of lower limb disease affecting gait.

### Ethical considerations-

This study did not require Research ethics approval or Health Research Authority clearance as per the Research and Innovation Department of the hospital. Gait assessment in these children is performed as routine clinical practice on every attendance to the MPS clinic. We accessed previously recorded gait analysis data which did not affect clinical practise. The data we extracted for assessment was handled as per Information governance guidelines. Informed verbal consent was taken as standard practice from the parent or guardian of each child before gait assessment. All procedures followed were in accordance with Helsinki declaration.

### Statistical analysis-

Statistical analysis was performed using SPSS software. Shapiro-Wilk test was used to determine that the data was not normally distributed. Comparison of median values of two related samples (pre-operative and post-operative measurements) was undertaken using the Wilcoxon signed rank test (non-parametric test). A two-tailed hypothesis with a *P*-value of ≤0.05 was deemed significant.

## Results

The pre-operative and post-operative gait assessment data (Table 2) of the 11 patients were analysed.

**Table 2:** Pre-operative and post-operative gait assessment data of all children included in the study

Patient no.	Pre-op walking distance (cm)	Post-op walking distance (cm)	Pre-op cadence (steps/unit time)	Post-op cadence (steps/unit time)	Pre-op velocity (cm/sec)	Post-op velocity (cm/sec)
1	1324.24	1325.53	94.1	98.8	74.2	70.4
2	1105.28	1344.74	140.1	133.5	80.2	99.8
3	2183.57	1636.79	114.8	109.4	88.9	99.4
4	1319.31	1308.11	128.9	139.5	118.1	118.4
5	1802.64	2019.6	145.7	138	95.2	145.2
6	1219.14	1749.55	129	126.6	124.8	99.8
7	766.26	1399.07	118.5	204.9	89	103.9
8	933.23	1523.57	152.7	114.5	99	70.9
9	893.01	1367.53	159.4	120.6	107.9	88.7
10	1670.63	1831.94	146.1	169.9	101.7	140.2
11	872.15	1135.77	103.3	146.5	68.2	106.6

**Fig. 4:**
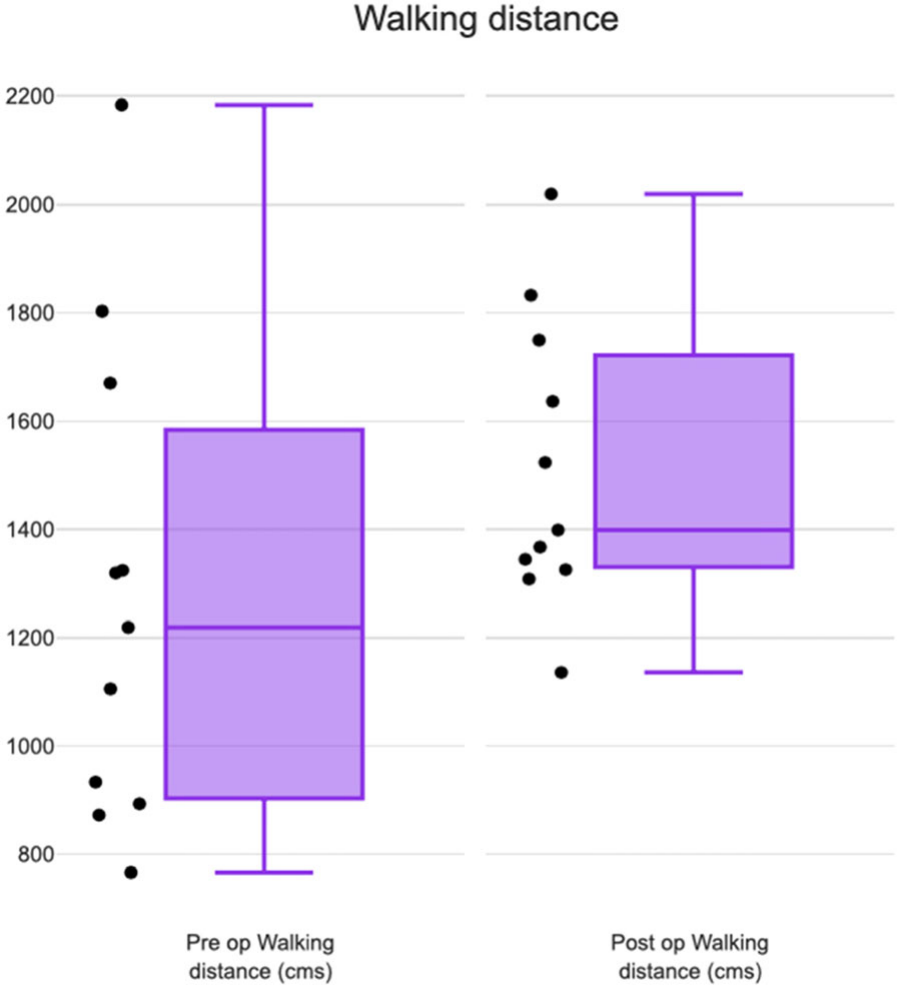
Box and Whisker plot comparing pre-operative and post-operative walking distance (cms)

Box and whisker plot was used to analyse distributional characteristics of the group of scores and the level of scores. The most significant observations were made when pre-operative and post-operative walking distance (Fig. 4) was compared. The median (dark purple line inside the box) or middle quartile of preop walking distance was 1219.14 cms and showed a significant increase to 1399.07cms postoperatively. The inter-quartile range is represented by the middlebox containing the middle 50% of the scores. The preop inter-quartile range was equally divided by the median, while the postoperative inter-quartile range was unequally divided by the median. The postop interquartile range showed a significant overall increase. The data points in the graph denotes 7 out of 11 patients clustered within the interquartile range postoperatively. It is also to be noted that 5 out of 11 patients clustered close to the median postoperatively, while they were widely distributed in relation to the median pre-operatively. The visible shortening of the postop inter-quartile range box with clustering of data points close to the median is reflected by statistically significant increase of walking distance postoperatively (*p* = 0.05, Wilcoxon signed rank). Walking distance increased by a mean of 232.06 cm post-operatively. The outliers in the lower whisker and upper whisker remained far from the median but showed proportionate improvement postoperatively.

**Fig. 5:**
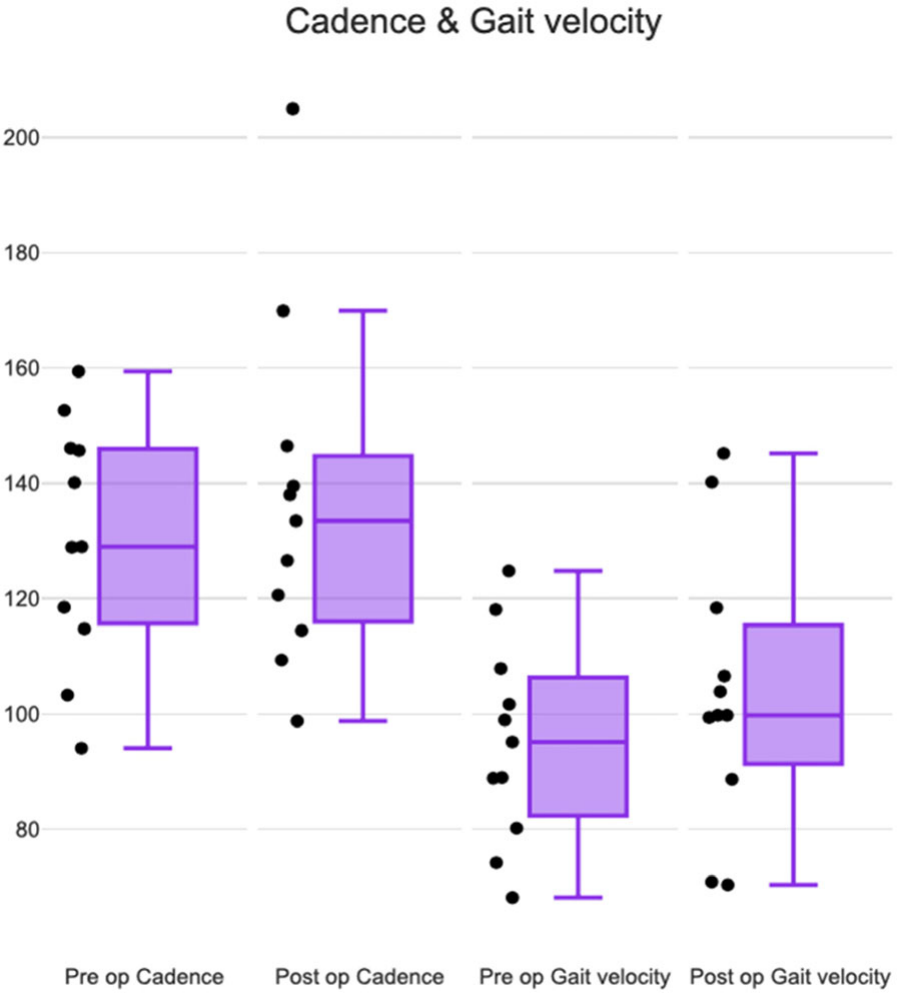
Box and Whisker plot comparing pre-operative and post-operative cadence (steps/min) and gait velocity (cms/sec)

The median of pre-op cadence was 129 and post-op cadence was 133.5. There was a marginal increase in cadence post-operatively (Fig. 5). There was no clustering of data points in the interquartile range. Change in post-op cadence was not statistically significant (*p*-value 0.79, Wilcoxon signed rank).

Gait velocity also showed a marginal increase post-operatively (Fig. 5) and therefore was similar to cadence on the box and whisker plot. The median of pre-op gait velocity was 95.2 cms/sec and post-op gait velocity was 99.8 cms/sec. Gait velocity increased by 8.73 cms/sec postoperatively and was not statistically significant (*p*-value 0.32, Wilcoxon Signed Rank).

## Discussion

Hurler syndrome was first described to predominantly involve the skeletal system [9]. Dysostosis multiplex refers to the complex of orthopaedic abnormalities resulting due to defective membranous and endochondral growth [10]. The effect of these orthopaedic abnormalities on locomotor ability of the affected child is profound. Children with Mucopolysaccharidosis 1 have below average gross motor abilities [11] and delayed maturation of gait [12]. Orthopaedic abnormalities including thoracolumbar kyphosis in this group can be progressive and surgical intervention may be justified. Thoracolumbar kyphosis with a Cobb’s angle of more than 40 degrees in children with Hurler syndrome is most likely to progress [13]. Our justification for spinal surgical intervention in this cohort of patients is based on clinical practise guidelines by International consensus procedure developed using a modified Delphi approach [7] with the eventual goal of treatment is to maintain or improve the quality of life.

Anaesthesia in MPS patient is complicated and requires expertise of Specialized Paediatric Anaesthetist with support of a multi disciplinary team including Otorhinolaryngologist and Intensivist. A few of the serious complications which can occur during and after surgery are inability to ventilate or intubate, temporary airway obstruction, complete airway obstruction, post intubation problems, stridor, lower airway collapse and need for reintubation or tracheostomy [14]. Odontoid hypoplasia and Atlantoaxial instability can cause spinal cord injury during manipulation of cervical spine at intubation in MPS type 4 and rarely in MPS type 6 patients. Thoracic cage abnormality, hepatosplenomegaly or neuromuscular causes can lead to reduced excursion of diaphragm which in turn can result in restrictive pulmonary disease [15–17]. Restrictive pulmonary disease along with airway obstruction can lead to cor pulmonale and respiratory failure [18]. The need for spinal intervention in our group of patients was decided by serial clinical and radiological assessments of the thoracolumbar kyphosis. A progressively worsening kyphosis, stiffening or irreducible curvature and threatened or imminent spinal cord compression were some of our indications for spinal surgery in selected patients.

There are seven types of Mucopolysaccharidosis and three subtypes of Mucopolysaccharidosis 1 (A-Hurler syndrome, B-Hurler-Scheie syndrome and C-Scheie syndrome). This study is the first attempt to assess functional outcome after Spinal deformity correction in patients with MPS of any subtype. Data obtained from GAITRite™ electronic walkway assessed spatio-temporal parameters of gait in this group of children before and after correction of thoracolumbar kyphosis. GAITRite™ walkway has been proven to have good reliability with immediate re-test, 2 week re-test and Intraclass correlation coefficients > 0.75 for measuring spatio-temporal parameters of gait [19] along with strong concurrent validity [20].

The nearest pre-operative and post-operative gait assessment data within 9 to 24 months of the index surgery were used for analysis. Children were found to be recovering from the index spinal surgery when assessed within 9 months of surgery. Therefore, time was given for the child to recover from the spinal surgery and then gait assessment was done. After 24 months there was a possibility of deterioration of gait due to the progression of disease in the hip and knee joints affecting global locomotor ability. Physiological maturation of gait is another potential confounding variable in growing children. Gait in the MPS study group may be expected to mature more slowly than the unaffected child. Restricting the post op assessment time frame to 9 months to 24 months helps us identify changes in the gait which can be attributed to spinal surgery and not due to physiological development. Gait analysis data of one patient alone was obtained 35 months after index spine surgery as that was the only available post-operative assessment data for that patient.

Walking distance is a good indicator of the overall ability of bipedal locomotion to propel the body from point A to point B. Walking distance or distance travelled on the walkway is measured in centimetres on the horizontal axis from heel centre of first foot print to the heel centre of last foot print [8]. This parameter is not affected by the disease process in the joints of the lower extremities or individual footfalls. Parameters such as stance phase can be affected if the disease process has resulted in antalgic gait from an affected hip joint. Similarly, step length can be recorded as a negative value if the child fails to bring the landing foot heel point forwards of the stationary foot making parameters of individual footfalls unreliable. Nine out of eleven patients in the study group exhibited a statistically significant increase in walking distance postoperatively (mean postoperative increase of 232.06 cms, *P*-value 0.05). There is no literature evidence to standardize normal walking distance according to age in this cohort.

Cadence is calculated as the number of steps taken per minute. Reducing cadence in a growing child is an indicator of maturing gait [21]. All the children in our study were assessed within 9 to 24 months from the index spine surgery, which was a cross-sectional analysis in a surgical cohort and not a linear assessment of gait maturation. We observed that there was a trend towards reduction of cadence postoperatively in six out of the eleven patients in the study group, which was not statistically significant (*P*-value 0.79). Therefore, physiological development did not have significant impact on gait during our assessment time frame postoperatively.

Gait velocity was calculated on the walkway after dividing the distance travelled by ambulation time. Measuring gait velocity reflected the ability or the ease with which the children walked after Spinal surgery. Gait velocity in our study increased in seven out of the eleven patients postoperatively. However, the mean increase in gait velocity by 8.73 cms/sec postoperatively was also not statistically significant (*P*-value 0.32). Cadence and gait velocity showed only a marginal improvement postoperatively.

Sagittal spinal curvatures help in maintaining global sagittal spinal balance and aid bipedal locomotion. A balanced spine requires minimal muscular effort to maintain an upright posture. Thoracolumbar kyphosis alters spinal biomechanics by resulting in an anterior shift of the truncal mass with increased flexion moment arm. An increased sagittal spinal curvature with increased flexion moment arm alters physiological loading of compression and shear forces across spinal segments [22]. Thoracolumbar kyphosis renders the normally strong dorsal extensor paraspinal musculature weak, affecting the ability to maintain an upright posture. The anterior truncal shift stretches the dorsal extensor paraspinal musculature beyond a point where the length-tension relationship is altered [23, 24].

Compensatory effect of sagittal spinal deformity is commonly observed in the neck and in the lower limbs. A backward tilt of the pelvis, flexion of the knees and dorsiflexion of the ankles are some of those seen in the lower extremity. The effort required for the lower limb to function in conjunction with the compensatory mechanism results in early fatigue with poor exercise tolerance. Children in this cohort have joints in lower extremities affected by the same disease process, adding to the burden of poor gait. Therefore, changes in sagittal spinal balance and poor lower limb function can both affect gait. Surgery to correct thoracolumbar kyphosis in our cohort is an attempt to restore sagittal spinal alignment which in turn has shown a functional improvement of gait. The reported improvement of gait in our study was attributed to restoration of sagittal spinal alignment as none of the children in the study group had any intervention to address the disease process in the joints of lower extremities within the post-operative assessment timeframe. Three children in our study group had hemiepiphysiodesis of knee joint done more than year before the index spinal surgical intervention. This will not have any impact on our study as we have taken the nearest pre-operative gait assessment as baseline.

Spine is the first component to get activated during gait and lumbar spine is the key component which drives the pelvis. EMG studies have shown early truncal muscle activation anticipating propulsion and gait initiation [25–27] proving that functioning of lower limbs follow spinal motion during gait. We did not use the standard 6 min walk test as the joints of the lower limbs were affected by the same disease process which may result in early fatigue. Gaitrite walkway is a validated tool [19, 20] in assessing spatiotemporal parameters of gait and we believe walking distance of one minute better reflects gait efficiency before lower limb fatigue sets in.

The process of maturation of gait in children with Mucopolysaccharidosis 1 is delayed when compared to normally developing children [11]. Improvement in cadence postoperatively was not statistically significant proving that physiological maturation had minimal effect on gait in the post-operative assessment timeframe. Therefore, the trend towards improved gait observed within the postoperative window (9 to 24 months) was due to surgical correction of the spinal deformity rather than developmental maturation of gait.

A limitation of our study was small sample size. Eleven patients from a single sub type (MPS1) is a large number, but remains a statistically small sample size. Studies with small sample size have a higher margin of Type 2 error and can over estimate the magnitude of association. However, we did manage to find out a statistically significant result (walking distance *p*-value 0.05) in one of the parameter analysed. Therefore, the positive association between spinal surgery to correct thoracolumbar kyphosis and gait should be considered a surrogate end point to design further multi centre studies with larger sample size. A larger sample size may yield statistically significant results in other parameters as well,

However, we do reflect the population of the largest centre for treatment of severe MPS1 in Europe and multi centre studies of surgical outcomes have proven challenging due to the variability in indication and procedure used in this group.

## Conclusion

The results of our study suggest that children with MPS1 who had Surgical correction of thoracolumbar kyphosis exhibited a trend towards improved gait after surgery. There was a statistically significant increase in the walking distance post-operatively with a trend towards improved cadence and gait velocity. The limitation of 9 to 24 months in the post-operative period ensured that our gait assessment was not affected by the physiological changes in gait or the disease process in joints of lower extremities.

Spinal surgery to correct thoracolumbar kyphosis or any orthopaedic intervention in this group of patients should aim to preserve or improve quality of life. Radiological outcome alone is insufficient to assess improvement following surgery and functional outcome should play a key role following surgery. Functional parameter such as gait can have a significant impact on the quality of life in this group of patients. We do not have a disease specific validated tool to assess quality of life in MPS, factoring in the varying levels of intellectual development across the sub types. Therefore, this study is a small step in the journey to develop a tool to assess functional ability following spinal surgery in children with MPS 1.

## Abbreviations

MPS: Mucopolysaccharidosis
MR: Magnetic resonance imaging
